# (*E*)-2-(4-Methoxy­phen­yl)-*N*-(2-pyrid­yl)-3-(2-pyridylamino)acrylamide

**DOI:** 10.1107/S1600536809007089

**Published:** 2009-03-06

**Authors:** Zhu-Ping Xiao, Xiao-Chun Peng, Ying-Chun Wang

**Affiliations:** a College of Chemistry & Chemical Engineering, Jishou University, Jishou 416000, People’s Republic of China

## Abstract

In the title compound, C_20_H_18_N_4_O_2_, the amino­acrylamide group makes a dihedral angles of 4.0 (1)° with the amino-bound pyridyl ring and 15.66 (12)° with the amide-bound pyridyl ring. The dihedral angle between the amino­acrylamide group and the pendant 4-methoxy­phenyl group is 71.22 (9)°. In the crystal structure, N—H⋯N hydrogen bonds and C—H⋯O and C—H⋯N inter­actions help to establish the packing. Intra­molecular C—H⋯O and C—H⋯(N,O) contacts also occur.

## Related literature

For background to the anti­bacteriological activity of enamines, see: Xiao *et al.* (2007 ▶, 2008 ▶).
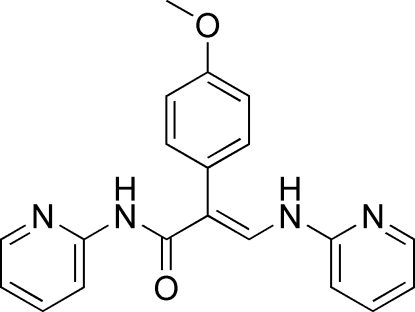


## Experimental

### 

#### Crystal data

C_20_H_18_N_4_O_2_
*M* *_r_* = 346.38
Monoclinic, 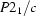

*a* = 11.546 (2) Å
*b* = 12.148 (2) Å
*c* = 14.006 (3) Å
β = 113.74 (3)°
*V* = 1798.3 (6) Å^3^
*Z* = 4
Mo *K*α radiation
μ = 0.09 mm^−1^
*T* = 293 K
0.30 × 0.10 × 0.10 mm

#### Data collection

Enraf–Nonius CAD-4 diffractometer
Absorption correction: ψ scan (North *et al.*, 1968 ▶) *T*_min_ = 0.975, *T*_max_ = 0.992
3523 measured reflections
3523 independent reflections
1452 reflections with *I* > 2σ(*I*)
3 standard reflections every 200 reflections intensity decay: none

#### Refinement

*R*[*F* ^2^ > 2σ(*F* ^2^)] = 0.083
*wR*(*F* ^2^) = 0.200
*S* = 1.04
3523 reflections
235 parameters
H-atom parameters constrained
Δρ_max_ = 0.16 e Å^−3^
Δρ_min_ = −0.16 e Å^−3^

### 

Data collection: *CAD-4 Software* (Enraf–Nonius, 1989 ▶); cell refinement: *CAD-4 Software*; data reduction: *XCAD4* (Harms & Wocadlo, 1995 ▶); program(s) used to solve structure: *SHELXS97* (Sheldrick, 2008 ▶); program(s) used to refine structure: *SHELXL97* (Sheldrick, 2008 ▶); molecular graphics: *SHELXTL* (Sheldrick, 2008 ▶); software used to prepare material for publication: *SHELXL97*.

## Supplementary Material

Crystal structure: contains datablocks global, I. DOI: 10.1107/S1600536809007089/hb2919sup1.cif

Structure factors: contains datablocks I. DOI: 10.1107/S1600536809007089/hb2919Isup2.hkl

Additional supplementary materials:  crystallographic information; 3D view; checkCIF report

## Figures and Tables

**Table 1:** Hydrogen-bond geometry (Å, °)

*D*—H⋯*A*	*D*—H	H⋯*A*	*D*⋯*A*	*D*—H⋯*A*
N1—H1*A*⋯N4^i^	0.86	2.25	3.079 (5)	163
C9—H9*A*⋯O2	0.93	2.33	2.718 (5)	104
C9—H9*A*⋯N2	0.93	2.42	2.754 (6)	101
C17—H17*A*⋯O2	0.93	2.27	2.850 (5)	120
C11—H11*A*⋯N4^i^	0.93	2.62	3.396 (6)	141
C14—H14*A*⋯O2^ii^	0.93	2.59	3.378 (5)	143

Symmetry codes: (i) 
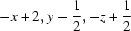
; (ii) 
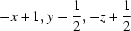
.

**Fig. 1.**
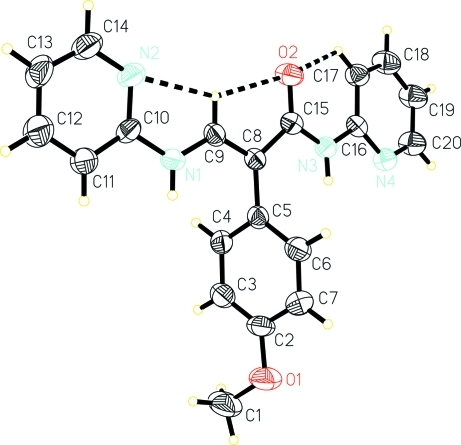
The molecular structure of (I) with displacement ellipsoids for the non-hydrogen atoms drawn at the 30% probability level.

## supplementary crystallographic information

### Comment

An enamine, a tautomer of a Schiff base, shows a high similarity to the corresponding Schiff base in chemical structure which shows diverse biological activities. Our recent work affirmed that enamine, like Schiff base, exhibited high antibacterial activity (Xiao *et al.*, 2007, 2008). We herein report the crystal structure of the title compound, (I).

As shown in Fig. 1, the title compound is an enamine containing a functional group of amide moiety. The title compound consists of an aminoacrylamide moiety and three aromatic ring fragments. The aminoacrylamide moiety, C8, C9, C15, N1, N3 and O2, is nearly coplanar with a mean deviation of 0.023 Å, defined as plane I; C10 to C14 and N2 forms a plane with a mean deviation of 0.005 Å, defined as plane II; C16 to C20 and N4 forms a plane with a mean deviation of 0.008 Å, defined as plane III; C2 to C7 forms the fourth plane with a mean deviation of 0.005 Å, defined as plane IV. Plane II, plane III and plane IV make a dihedral angle of 4.018 (8), 15.66 (12) and 71.22 (9) ° with plane I, respectively. The bond distance C8—C9 [1.359 (5) Å] falls in the range of a typical double bond, and C9—N1 bond [1.352 (4) Å] is shorter than the standard C—N single bond (1.48 Å), but longer than a C—N double bond (1.28 Å). This clearly indicates that the p orbital of N1 is conjugated with the π molecular orbital of C8—C9 double bond. For the same reason, we speculate that the p orbital of N1 is also conjugated with pyridyl group (plane II) and the p orbital of N3 is conjugated with both pyridyl group (plane III) and carboxyl group (C15=O2). All other double bonds and single bonds in the molecule fall in normal range of bond lengths.

In the crystal of (I), the structure is stabilized by intermolecular interactions including hydrogen bond N1—H1A···N4, C11—H11A···N4 and C14—H14A···O2; details of hydrogen-bond geometry are given in Table 1.

### Experimental

Equimolar quantities (6 mmol) of 2-(4-methoxyphenyl)-3-oxo-*N*-(pyridin-2-yl)propanamide (1.62 g) and 2-aminobenzenamine (0.56 g) in absolute alcohol (18 ml) were heated at 344–354 K for 2 h. The excess solvent was removed under reduced pressure. The residue was purified by a flash chromatography with EtOAc-petrolum ether to afford two fractions. The first fraction gave a *Z*-isomer, and the second fraction, after partial solvent evaporation, furnished colourless blocks of (I) suitable for single-crystal structure determination.

### Refinement

All H atoms were placed in geometrically idealized positions (C—H = 0.93–0.96Å, N—H = 0.86Å) and refined as riding with *U*_iso_(H) = 1.2U_eq_(C,N) or 1.5U_eq_(methyl C).

### Crystal data

**Table:**

C_20_H_18_N_4_O_2_	*F*(000) = 728
*M**_r_* = 346.38	*D*_x_ = 1.279 Mg m^−^^3^
Monoclinic, *P*2_1_/*c*	Mo *K*α radiation, λ = 0.71073 Å
Hall symbol: -P 2ybc	Cell parameters from 25 reflections
*a* = 11.546 (2) Å	θ = 9–12°
*b* = 12.148 (2) Å	µ = 0.09 mm^−^^1^
*c* = 14.006 (3) Å	*T* = 293 K
β = 113.74 (3)°	Block, colorless
*V* = 1798.3 (6) Å^3^	0.30 × 0.10 × 0.10 mm
*Z* = 4	

### Data collection

**Table:**

Enraf–Nonius CAD-4 diffractometer	1452 reflections with *I* > 2σ(*I*)
Radiation source: fine-focus sealed tube	*R*_int_ = 0.0000
graphite	θ_max_ = 26.0°, θ_min_ = 1.9°
ω/2θ scans	*h* = −14→13
Absorption correction: ψ scan (North *et al.*, 1968)	*k* = 0→14
*T*_min_ = 0.975, *T*_max_ = 0.992	*l* = 0→17
3523 measured reflections	3 standard reflections every 200 reflections
3523 independent reflections	intensity decay: none

### Refinement

**Table:**

Refinement on *F*^2^	Primary atom site location: structure-invariant direct methods
Least-squares matrix: full	Secondary atom site location: difference Fourier map
*R*[*F*^2^ > 2σ(*F*^2^)] = 0.083	Hydrogen site location: inferred from neighbouring sites
*wR*(*F*^2^) = 0.200	H-atom parameters constrained
*S* = 1.04	*w* = 1/[σ^2^(*F*_o_^2^) + (0.0712*P*)^2^] where *P* = (*F*_o_^2^ + 2*F*_c_^2^)/3
3523 reflections	(Δ/σ)_max_ < 0.001
235 parameters	Δρ_max_ = 0.16 e Å^−^^3^
0 restraints	Δρ_min_ = −0.16 e Å^−^^3^

### Special details

**Table:**

Geometry. All e.s.d.'s (except the e.s.d. in the dihedral angle between two l.s. planes) are estimated using the full covariance matrix. The cell e.s.d.'s are taken into account individually in the estimation of e.s.d.'s in distances, angles and torsion angles; correlations between e.s.d.'s in cell parameters are only used when they are defined by crystal symmetry. An approximate (isotropic) treatment of cell e.s.d.'s is used for estimating e.s.d.'s involving l.s. planes.
Refinement. Refinement of *F*^2^ against ALL reflections. The weighted *R*-factor *wR* and goodness of fit *S* are based on *F*^2^, conventional *R*-factors *R* are based on *F*, with *F* set to zero for negative *F*^2^. The threshold expression of *F*^2^ > σ(*F*^2^) is used only for calculating *R*-factors(gt) *etc*. and is not relevant to the choice of reflections for refinement. *R*-factors based on *F*^2^ are statistically about twice as large as those based on *F*, and *R*- factors based on ALL data will be even larger.

### Fractional atomic coordinates and isotropic or equivalent isotropic displacement parameters (Å^2^)

**Table:**

	*x*	*y*	*z*	*U*_iso_*/*U*_eq_	
N1	0.8670 (3)	0.3253 (3)	0.3474 (3)	0.0628 (10)	
H1A	0.9483	0.3244	0.3725	0.075*	
O1	1.3868 (3)	0.5123 (3)	0.3652 (3)	0.1153 (14)	
C1	1.4551 (5)	0.4266 (5)	0.3437 (5)	0.137 (3)	
H1B	1.5434	0.4450	0.3718	0.206*	
H1C	1.4251	0.4168	0.2696	0.206*	
H1D	1.4432	0.3596	0.3750	0.206*	
O2	0.6560 (2)	0.5617 (3)	0.1480 (2)	0.0756 (9)	
N2	0.6813 (3)	0.2469 (3)	0.3418 (3)	0.0752 (11)	
C2	1.2592 (4)	0.5024 (5)	0.3320 (4)	0.0820 (15)	
N3	0.8252 (3)	0.6425 (3)	0.1344 (2)	0.0591 (9)	
H3A	0.9063	0.6471	0.1648	0.071*	
C3	1.1876 (4)	0.4216 (4)	0.2676 (4)	0.0764 (13)	
H3B	1.2254	0.3682	0.2420	0.092*	
N4	0.8483 (3)	0.7805 (3)	0.0328 (3)	0.0635 (9)	
C4	1.0576 (4)	0.4195 (3)	0.2404 (3)	0.0648 (11)	
H4B	1.0096	0.3645	0.1958	0.078*	
C5	0.9974 (4)	0.4961 (3)	0.2771 (3)	0.0568 (11)	
C6	1.0728 (4)	0.5774 (4)	0.3409 (3)	0.0743 (13)	
H6A	1.0356	0.6313	0.3665	0.089*	
C7	1.2003 (4)	0.5811 (4)	0.3678 (4)	0.0874 (15)	
H7A	1.2479	0.6373	0.4107	0.105*	
C8	0.8599 (3)	0.4900 (3)	0.2477 (3)	0.0545 (10)	
C9	0.8061 (4)	0.4077 (3)	0.2814 (3)	0.0583 (11)	
H9A	0.7183	0.4081	0.2566	0.070*	
C10	0.8054 (4)	0.2412 (3)	0.3773 (3)	0.0602 (11)	
C11	0.8757 (4)	0.1591 (4)	0.4414 (4)	0.0797 (15)	
H11A	0.9634	0.1585	0.4652	0.096*	
C12	0.8136 (5)	0.0787 (4)	0.4691 (4)	0.1026 (18)	
H12A	0.8588	0.0216	0.5122	0.123*	
C13	0.6855 (5)	0.0816 (4)	0.4340 (4)	0.0955 (17)	
H13A	0.6416	0.0268	0.4518	0.115*	
C14	0.6240 (5)	0.1666 (4)	0.3725 (4)	0.0884 (16)	
H14A	0.5365	0.1697	0.3499	0.106*	
C15	0.7711 (4)	0.5672 (3)	0.1737 (3)	0.0537 (10)	
C16	0.7672 (4)	0.7143 (3)	0.0506 (3)	0.0559 (10)	
C17	0.6370 (4)	0.7165 (4)	−0.0105 (3)	0.0671 (12)	
H17A	0.5812	0.6716	0.0045	0.080*	
C18	0.5952 (5)	0.7878 (4)	−0.0932 (4)	0.0870 (15)	
H18A	0.5094	0.7909	−0.1362	0.104*	
C19	0.6782 (5)	0.8542 (4)	−0.1129 (4)	0.0860 (16)	
H19A	0.6505	0.9018	−0.1697	0.103*	
C20	0.8027 (4)	0.8489 (4)	−0.0471 (4)	0.0811 (14)	
H20A	0.8588	0.8961	−0.0591	0.097*	

### Atomic displacement parameters (Å^2^)

**Table:**

	*U*^11^	*U*^22^	*U*^33^	*U*^12^	*U*^13^	*U*^23^
N1	0.0472 (19)	0.066 (2)	0.072 (2)	0.0045 (18)	0.0203 (17)	0.011 (2)
O1	0.056 (2)	0.122 (3)	0.161 (4)	0.003 (2)	0.036 (2)	0.026 (3)
C1	0.066 (3)	0.138 (6)	0.215 (7)	0.023 (4)	0.064 (4)	0.051 (5)
O2	0.0566 (18)	0.090 (2)	0.082 (2)	0.0110 (16)	0.0303 (16)	0.0173 (18)
N2	0.055 (2)	0.079 (3)	0.086 (3)	−0.011 (2)	0.023 (2)	0.018 (2)
C2	0.046 (3)	0.091 (4)	0.100 (4)	0.005 (3)	0.020 (3)	0.032 (3)
N3	0.0523 (19)	0.056 (2)	0.059 (2)	0.0026 (17)	0.0119 (17)	0.0122 (18)
C3	0.070 (3)	0.073 (3)	0.092 (4)	0.011 (3)	0.038 (3)	0.015 (3)
N4	0.062 (2)	0.070 (2)	0.056 (2)	0.000 (2)	0.0210 (18)	0.009 (2)
C4	0.065 (3)	0.056 (3)	0.067 (3)	0.000 (2)	0.020 (2)	0.002 (2)
C5	0.059 (2)	0.051 (3)	0.054 (3)	0.005 (2)	0.015 (2)	0.005 (2)
C6	0.064 (3)	0.077 (3)	0.075 (3)	0.009 (3)	0.021 (2)	−0.004 (3)
C7	0.066 (3)	0.083 (4)	0.094 (4)	−0.007 (3)	0.013 (3)	−0.008 (3)
C8	0.052 (2)	0.050 (3)	0.057 (3)	0.006 (2)	0.0169 (19)	0.003 (2)
C9	0.056 (2)	0.050 (2)	0.059 (3)	0.007 (2)	0.012 (2)	0.000 (2)
C10	0.057 (3)	0.063 (3)	0.057 (3)	−0.015 (2)	0.019 (2)	0.004 (2)
C11	0.066 (3)	0.080 (3)	0.084 (3)	−0.001 (3)	0.021 (3)	0.034 (3)
C12	0.093 (4)	0.098 (4)	0.101 (4)	−0.008 (3)	0.023 (3)	0.040 (4)
C13	0.095 (4)	0.081 (4)	0.095 (4)	−0.022 (3)	0.023 (3)	0.031 (3)
C14	0.067 (3)	0.096 (4)	0.098 (4)	−0.021 (3)	0.030 (3)	0.014 (3)
C15	0.055 (2)	0.059 (3)	0.047 (2)	0.004 (2)	0.021 (2)	−0.003 (2)
C16	0.057 (2)	0.056 (3)	0.054 (3)	0.010 (2)	0.022 (2)	0.003 (2)
C17	0.060 (3)	0.073 (3)	0.062 (3)	0.003 (2)	0.017 (2)	0.009 (3)
C18	0.066 (3)	0.106 (4)	0.068 (3)	0.016 (3)	0.005 (3)	0.017 (3)
C19	0.074 (3)	0.103 (4)	0.077 (4)	0.017 (3)	0.025 (3)	0.039 (3)
C20	0.075 (3)	0.097 (4)	0.071 (3)	0.006 (3)	0.029 (3)	0.023 (3)

### Geometric parameters (Å, °)

**Table:**

N1—C9	1.352 (4)	C5—C8	1.472 (5)
N1—C10	1.402 (5)	C6—C7	1.366 (6)
N1—H1A	0.8600	C6—H6A	0.9300
O1—C2	1.360 (5)	C7—H7A	0.9300
O1—C1	1.409 (6)	C8—C9	1.359 (5)
C1—H1B	0.9600	C8—C15	1.466 (5)
C1—H1C	0.9600	C9—H9A	0.9300
C1—H1D	0.9600	C10—C11	1.368 (5)
O2—C15	1.231 (4)	C11—C12	1.358 (6)
N2—C10	1.315 (5)	C11—H11A	0.9300
N2—C14	1.343 (5)	C12—C13	1.358 (6)
C2—C3	1.365 (6)	C12—H12A	0.9300
C2—C7	1.379 (6)	C13—C14	1.348 (6)
N3—C15	1.344 (5)	C13—H13A	0.9300
N3—C16	1.398 (5)	C14—H14A	0.9300
N3—H3A	0.8600	C16—C17	1.400 (5)
C3—C4	1.393 (5)	C17—C18	1.368 (6)
C3—H3B	0.9300	C17—H17A	0.9300
N4—C20	1.321 (5)	C18—C19	1.364 (6)
N4—C16	1.332 (5)	C18—H18A	0.9300
C4—C5	1.379 (5)	C19—C20	1.361 (6)
C4—H4B	0.9300	C19—H19A	0.9300
C5—C6	1.380 (5)	C20—H20A	0.9300
			
C9—N1—C10	123.8 (3)	N1—C9—C8	126.7 (4)
C9—N1—H1A	118.1	N1—C9—H9A	116.7
C10—N1—H1A	118.1	C8—C9—H9A	116.7
C2—O1—C1	119.0 (5)	N2—C10—C11	123.7 (4)
O1—C1—H1B	109.5	N2—C10—N1	117.1 (4)
O1—C1—H1C	109.5	C11—C10—N1	119.2 (4)
H1B—C1—H1C	109.5	C12—C11—C10	118.0 (4)
O1—C1—H1D	109.5	C12—C11—H11A	121.0
H1B—C1—H1D	109.5	C10—C11—H11A	121.0
H1C—C1—H1D	109.5	C11—C12—C13	120.1 (5)
C10—N2—C14	116.3 (4)	C11—C12—H12A	120.0
O1—C2—C3	125.0 (5)	C13—C12—H12A	120.0
O1—C2—C7	116.1 (5)	C14—C13—C12	117.9 (5)
C3—C2—C7	118.8 (4)	C14—C13—H13A	121.0
C15—N3—C16	128.6 (4)	C12—C13—H13A	121.0
C15—N3—H3A	115.7	N2—C14—C13	124.0 (5)
C16—N3—H3A	115.7	N2—C14—H14A	118.0
C2—C3—C4	119.6 (5)	C13—C14—H14A	118.0
C2—C3—H3B	120.2	O2—C15—N3	122.9 (4)
C4—C3—H3B	120.2	O2—C15—C8	122.5 (4)
C20—N4—C16	117.9 (4)	N3—C15—C8	114.6 (4)
C5—C4—C3	122.4 (4)	N4—C16—N3	113.5 (3)
C5—C4—H4B	118.8	N4—C16—C17	122.5 (4)
C3—C4—H4B	118.8	N3—C16—C17	124.0 (4)
C4—C5—C6	116.3 (4)	C18—C17—C16	117.1 (4)
C4—C5—C8	120.7 (4)	C18—C17—H17A	121.4
C6—C5—C8	123.0 (4)	C16—C17—H17A	121.4
C7—C6—C5	122.0 (4)	C19—C18—C17	120.6 (4)
C7—C6—H6A	119.0	C19—C18—H18A	119.7
C5—C6—H6A	119.0	C17—C18—H18A	119.7
C6—C7—C2	120.8 (5)	C20—C19—C18	118.2 (5)
C6—C7—H7A	119.6	C20—C19—H19A	120.9
C2—C7—H7A	119.6	C18—C19—H19A	120.9
C9—C8—C15	115.4 (4)	N4—C20—C19	123.7 (5)
C9—C8—C5	122.2 (4)	N4—C20—H20A	118.1
C15—C8—C5	122.3 (4)	C19—C20—H20A	118.1
			
C1—O1—C2—C3	−8.5 (8)	N2—C10—C11—C12	0.5 (7)
C1—O1—C2—C7	171.2 (5)	N1—C10—C11—C12	−180.0 (4)
O1—C2—C3—C4	179.1 (4)	C10—C11—C12—C13	−0.4 (8)
C7—C2—C3—C4	−0.6 (7)	C11—C12—C13—C14	−0.6 (9)
C2—C3—C4—C5	−0.7 (7)	C10—N2—C14—C13	−1.6 (7)
C3—C4—C5—C6	1.5 (6)	C12—C13—C14—N2	1.7 (9)
C3—C4—C5—C8	−179.1 (4)	C16—N3—C15—O2	−10.3 (6)
C4—C5—C6—C7	−1.0 (6)	C16—N3—C15—C8	168.0 (4)
C8—C5—C6—C7	179.6 (4)	C9—C8—C15—O2	3.7 (6)
C5—C6—C7—C2	−0.3 (7)	C5—C8—C15—O2	179.2 (4)
O1—C2—C7—C6	−178.6 (4)	C9—C8—C15—N3	−174.7 (3)
C3—C2—C7—C6	1.2 (7)	C5—C8—C15—N3	0.8 (5)
C4—C5—C8—C9	68.4 (5)	C20—N4—C16—N3	177.8 (4)
C6—C5—C8—C9	−112.2 (5)	C20—N4—C16—C17	−1.4 (6)
C4—C5—C8—C15	−106.8 (4)	C15—N3—C16—N4	178.7 (4)
C6—C5—C8—C15	72.6 (5)	C15—N3—C16—C17	−2.1 (6)
C10—N1—C9—C8	−178.4 (4)	N4—C16—C17—C18	2.4 (6)
C15—C8—C9—N1	178.4 (4)	N3—C16—C17—C18	−176.8 (4)
C5—C8—C9—N1	2.9 (6)	C16—C17—C18—C19	−1.0 (7)
C14—N2—C10—C11	0.5 (7)	C17—C18—C19—C20	−1.2 (8)
C14—N2—C10—N1	−179.0 (4)	C16—N4—C20—C19	−1.0 (7)
C9—N1—C10—N2	−3.0 (6)	C18—C19—C20—N4	2.3 (8)
C9—N1—C10—C11	177.5 (4)		

### Hydrogen-bond geometry (Å, °)

**Table:**

*D*—H···*A*	*D*—H	H···*A*	*D*···*A*	*D*—H···*A*
N1—H1A···N4^i^	0.86	2.25	3.079 (5)	163
C9—H9A···O2	0.93	2.33	2.718 (5)	104
C9—H9A···N2	0.93	2.42	2.754 (6)	101
C17—H17A···O2	0.93	2.27	2.850 (5)	120
C11—H11A···N4^i^	0.93	2.62	3.396 (6)	141
C14—H14A···O2^ii^	0.93	2.59	3.378 (5)	143

Symmetry codes: (i) −*x*+2, *y*−1/2, −*z*+1/2; (ii) −*x*+1, *y*−1/2, −*z*+1/2.
